# Pregnancy‐Related Venous Thromboembolism Risk Perception and Prevention in Risk‐Averse Times—Significant Change Required: a Commentary

**DOI:** 10.1111/1471-0528.18229

**Published:** 2025-05-21

**Authors:** Laura A. Magee, Roopen Arya, Clare Boag, Luci Buxton, Matthew A. Coleman, Fizzah Jivraj, Rebecca Scott, Kayleigh Sheen, Sergio A. Silverio, Peter von Dadelszen

**Affiliations:** ^1^ Department of Women and Children's Health, School of Life Course & Population Sciences King's College London London UK; ^2^ King's Thrombosis Centre, Department of Haematological Medicine King's College Hospital NHS Foundation Trust London UK; ^3^ Family Health Clinical Board The Newcastle Upon Tyne Hospitals NHS Foundation Trust Newcastle‐upon‐Tyne UK; ^4^ Maternity Services, Elizabeth Garrett Anderson Wing University College Hospitals NHS Foundation Trust London UK; ^5^ Maternity Services, Princess Anne Hospital University Hospital Southampton NHS Foundation Trust Southampton UK; ^6^ School of Bioscience Education, Faculty of Life Sciences & Medicine King's College London London UK; ^7^ Maternity Services, Chelsea and Westminster Hospital Chelsea and Westminster Hospital NHS Foundation Trust London UK; ^8^ School of Social Sciences, College of Health, Science and Society University of the West of England UK; ^9^ Department of Psychology Institute of Population Health, University of Liverpool Liverpool UK

**Keywords:** venous thromobemolism, haematology, maternity services, risk perception

## Abstract

Between 2020 and 2022 in the United Kingdom (UK), there were 45 maternal deaths from venous thromboembolism (VTE), out of more than 2 million maternities. This occurred despite extensive risk assessment and prescribing of low molecular weight heparin (LMWH) thromboprophylaxis, alongside clinicians' overestimate of risk and commitment to the cause. Whilst every maternal death is a tragedy, the challenge ahead is immense—to identify, in an efficient and consistent way, those few women at risk of life‐threatening thrombosis, and then minimise that risk with a cost‐effective therapy that is acceptable to pregnant and postpartum women, and does not do more harm than good. We propose a way forward.

## Abstract 2

This article includes Author Insights, a video abstract available at: https://vimeo.com/1072760896.

## Graphical abstract

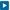

Venous thromboembolism (VTE) occurs in approximately 1:500 (0.2%) of all pregnancies; of those, approximately 1% are fatal (1:50 000 pregnancies). This makes VTE both a rare cause of maternal mortality and the single most common cause in the United Kingdom (UK) and Ireland, at 2.12 per 100 000 maternities [1]. Following a nadir in the VTE‐related maternal mortality rate during the 2017–2019 triennium, and despite extensive efforts to highlight VTE risk, objectify estimation of that risk, and offer thromboprophylaxis to those at increased risk [2], VTE‐related maternal mortality has been rising. Of note, however, is that the rise has been proportionate to the rise in overall, direct and indirect maternal mortality in the UK (Table 1).

**TABLE 1:** Maternal mortality rates (and 95% CI) in the UK and Ireland, by overlapping triennia (2016–2022).

Triennia	VTE‐related	% of previous triennium	Overall (direct and indirect)	% of previous triennium	All direct	Haemorrhage	All indirect
2016–2018	1.48 (1.02–2.07)	NA	9.71 (8.46–11.09)	NA	4.12 (3.32–5.05)	0.63 (0.34–1.05)	5.59 (4.66–6.66)
2017–2019	0.92 (0.56–1.42)	66%	8.79 (7.58–10.12)	91%	3.59 (2.84–4.48)	0.64 (0.35–1.08)	5.20 (4.28–6.25)
2018–2020	1.38 (0.92–1.98)	150%	10.90 (9.53–12.40)	121%	5.19 (4.26–6.26)	0.76 (0.44–1.24)	5.71 (4.73–6.83)
2019–2021	1.60 (1.10–2.24)	116%	11.66 (10.23–13.23)	107%	5.47 (4.51–6.57)	0.82 (0.48–1.32)	6.19 (5.17–7.36)
2020–2022	2.12 (1.53–2.86)	133%	13.56 (12.00–15.26)	116%	6.36 (5.32–7.56)	0.89 (0.53–1.40)	7.20 (6.08–8.46)

Abbreviations: CI, confidence interval; VTE, venous thromboembolism.

*Source:* MBRRACE [1].

VTE and VTE‐related death in pregnancy and postpartum are rare. The VTE‐related maternal mortality rate is comparable to the annualised risk of death from a motor vehicle accident in the UK. However, the perception of VTE‐related risk is (very) distorted. As part of early stakeholder engagement, we undertook a survey of Royal College of Obstetricians and Gynaecologists (RCOG) Green‐Top Guideline (GTG)‐37a users attending obstetric medicine and maternal‐fetal medicine conferences in the UK; of 142 clinician responders, many overestimated the risk of developing VTE (34%) or dying from it (68%). This may drive comfort with over‐diagnosis, over‐medicalisation, and over‐treatment, which are contributing to our current healthcare crisis [3].

Guidelines have been described by Kotaska as ‘mute’ on the absolute risks of VTE in pregnancy and postpartum, as well as absolute risk reductions associated with thromboprophylaxis, and the relevant numbers needed to treat for *benefit* (NNTB) or harm (NNTH) [4]. Whilst there are exceptions to this statement, as some guidelines cover absolute risks of VTE and VTE‐related harms [5], it must be acknowledged that the relevant data are often lacking, but needed. Discussion of absolute risk is a key principle of objective, shared decision‐making.

Values for NNTB and NNTH may be much higher than either women or care‐providers comprehend or estimate. For example, in our informal survey of VTE guideline users in the UK, the absolute risk of VTE that responders felt warranted thromboprophylaxis was 26% antenatally and 27% postnatally, levels not achieved even for women with prior VTE. Also, identifying women at increased risk of postpartum VTE using the RCOG 2015 guidelines and assuming a 50% effectiveness of low molecular weight heparin (LMWH), 1538 postpartum women would need to receive LMWH for ≥ 10 days postpartum to prevent one VTE. For comparison, the NNTB with magnesium sulphate (on average for 24 h) to prevent one seizure in pre‐eclampsia is 100 or 50 if one restricts treatment to women with more severe disease by Magpie trial standards. Also, with regards to the NNTH, the stated risks of LMWH are variable; the 2014 Canadian guidelines estimate these collectively to be < 1% [5], but a 2022 decision‐analytic model used estimates for major bleeding that were closer to 5% postpartum (even in unselected postpartum women) [6], and wound haematoma estimates (0.6%).

There are many VTE risk factors, most of which have weak associations with VTE and are highly likely to have been distorted by recall bias in some study designs. Risk assessment models (RAMs) have integrated these risk factors. A recent systematic review [7] identified 19 externally validated and one internally validated RAMs for use in pregnancy or postpartum. Most RAMs were found to lack rigorous development and evaluation, be at substantial risk of bias, and have highly variable predictive accuracy (usually sensitivity ≥ 80%, but specificity < 50%, particularly antenatally). In pregnancy, the most common models used (e.g., RCOG 2015) have assigned points to risk factors, based on their strength of association with VTE and treating them independently; however, this cannot account for the lack of independence in risk (e.g., between obesity and assisted reproductive technology, or between older age and higher BMI) or likely interactions between risk factors (e.g., obesity and mobility). Postnatally, multivariable models have modest predictive abilities. For example, the Sultan 2016 model has a C‐statistic of 0.70 (95% CI 0.67–0.73), a screen‐positive rate of 35% (to be offered thromboprophylaxis), and a detection rate of 68% for postnatal VTE [8]; assuming that LMWH halves VTE risk, this means that, only 35% of VTEs would be prevented using this RAM to identify women at risk and offer LMWH thromboprophylaxis.

The cost‐effectiveness of various thromboprophylaxis strategies with weight‐adjusted LMWH has been examined, based on net monetary benefit and quality‐adjusted life years [6]. The objective was to establish priorities for research, given the lack of relevant trial‐based evidence. Antenatally, only high‐risk women were considered, such as those with prior VTE or higher risk thrombophilias. There was no optimal strategy, from among those considered (i.e., antenatal plus postnatal thromboprophylaxis, postnatal thromboprophylaxis only, or neither antenatal nor postnatal thromboprophylaxis), but a trial was not considered feasible, based on prior attempts with poor recruitment and informative qualitative studies [6]. Postpartum, it was highly likely (≈90%) that no prophylaxis was the optimal strategy in the general maternity population postpartum, or those specifically who had given birth by Caesarean; this is very different to current levels of suggested thromboprophylaxis of up to 30% of patients [8]. Only in obese women (with BMI ≥ 30 kg/m^2^) was risk‐based thromboprophylaxis found to be potentially optimal, feasible, and worthy of future research. These analyses highlight the challenges of the low VTE event rate, even for women with risk factors with some of the strongest apparent relationships with VTE.

Clinical practice guidelines recommend VTE risk assessment (and treatment) based largely on low‐ or very low‐quality evidence [5], given the lack of relevant clinical trials [9], hampered by prohibitive sample size estimates and recruitment challenges that mandate strong international collaboration [10]. There is general agreement that risk assessment should be individualised, LMWH is the agent of choice, and thromboprophylaxis should start early in pregnancy when sufficient VTE risk (usually undefined) is identified. However, there is no account of factors which decrease risk (such as thrombophilia with no prior VTE, or prior oral contraception exposure and previous pregnancy without prior VTE), and there is inconsistency in which risk factors on their own pose a sufficient risk of VTE to warrant thromboprophylaxis.

Currently, we do not have decision aids to facilitate discussions between care‐providers and care‐users regarding antepnatal or postpartum thromboprophylaxis, which may have a negative impact on compliance with VTE prophylaxis, particularly postpartum [11]. However, the advanced work on evaluating women's decision‐making about antenatal thromboprophylaxis [12] suggests that women may be more VTE risk‐averse and accepting of LMWH than may be anticipated based on NNTB and NNTH. Similar findings were reported following a recent attempt to develop a decision aid for postpartum thromboprophylaxis among European women, who had a low absolute risk threshold for thromboprophylaxis, which they found acceptable at a postpartum VTE incidence of 0.2% (interquartile range: 0.1%–5%) when the bleeding risk of LMWH was 1% [13]. However, the wide interquartile range of risk threshold illustrates the spectrum of opinion observed and highlights the need to consider individual preferences.

## 1 Where Do We Go From Here?

There is an urgent need for a substantial modification to pregnancy‐ and postpartum‐related VTE risk assessment and management. This was noted in a new recommendation in the latest report from MBRRACE [1], which has repeatedly stated that the current VTE risk‐scoring systems (antenatally and postpartum) are difficult to apply consistently in practice. Healthcare providers who responded to our survey (as above) agreed; over 35% said that the current UK system is too complex, and almost 30% did not feel confident in using it to accurately assess VTE risk. Also, women are not adhering to thromboprophylaxis offered, in part or in whole, particularly postpartum, and LMWH is an unpleasant and costly medication, which, like all medication, has potential adverse effects that must be addressed to reach informed decisions.

An optimal RAM, for assessment antenatally and postpartum when VTE risk is higher, would account for clinical outcomes, costs, and quality of life, but none is available. This is unlikely to be achievable, at least in the near future, given the rarity of VTE, prevalent use of thromboprophylaxis, and absence of ultrasonographic and/or biomarkers, adjusted for personal characteristics (e.g., ethnicity) as they are for prenatal diagnosis or pre‐eclampsia prediction. There is the additional challenge of predicting antenatal events over a longer period of time (at least 6 months) than postnatal events (over 6 weeks). However, steps toward addressing this challenge could be made through objective, observational data on current risk factors and subsequent disease prevalence. Recently established, national maternal medicine networks have an opportunity to lead and support this development.

**FIGURE 1:**
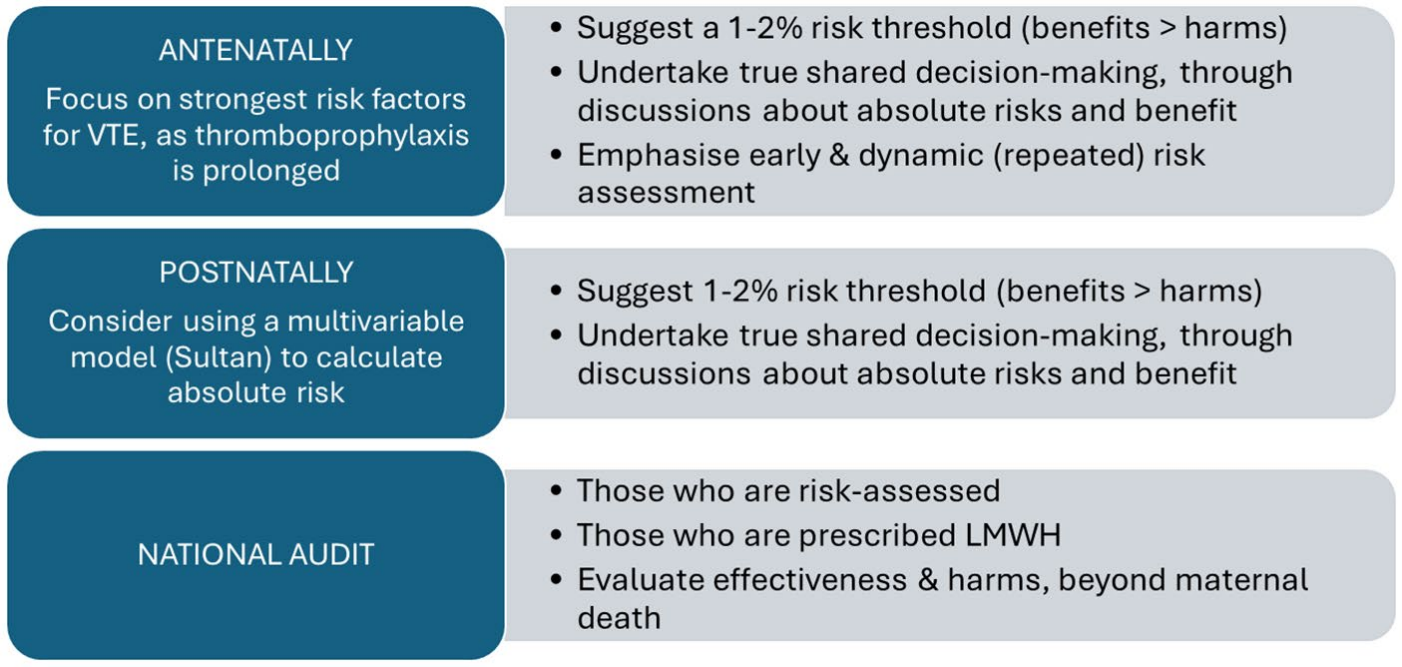
Potential strategy for VTE thromboprophylaxis in pregnancy and postpartum.

We outline a potential strategy in Figure 1. Antenatally, this involves focusing on the strongest VTE risk factors for which there is consensus that thromboprophylaxis is warranted, and setting an absolute VTE risk threshold for thromboprophylaxis (such as 1%–2%), to maximise the likely benefits and minimise potential harms. Postpartum, if a multivariable model were used to estimate the absolute risk of VTE, despite the models' stated limitations (as above), an estimated 50% risk reduction with LMWH could be used to estimate NNTB and NNTH for shared decision‐making with women, ideally with a formal evidence‐based decision aid. Accurate and consistent national data collection could quickly confirm the appropriateness of this new, absolute risk‐based threshold.

**FIGURE 2:**
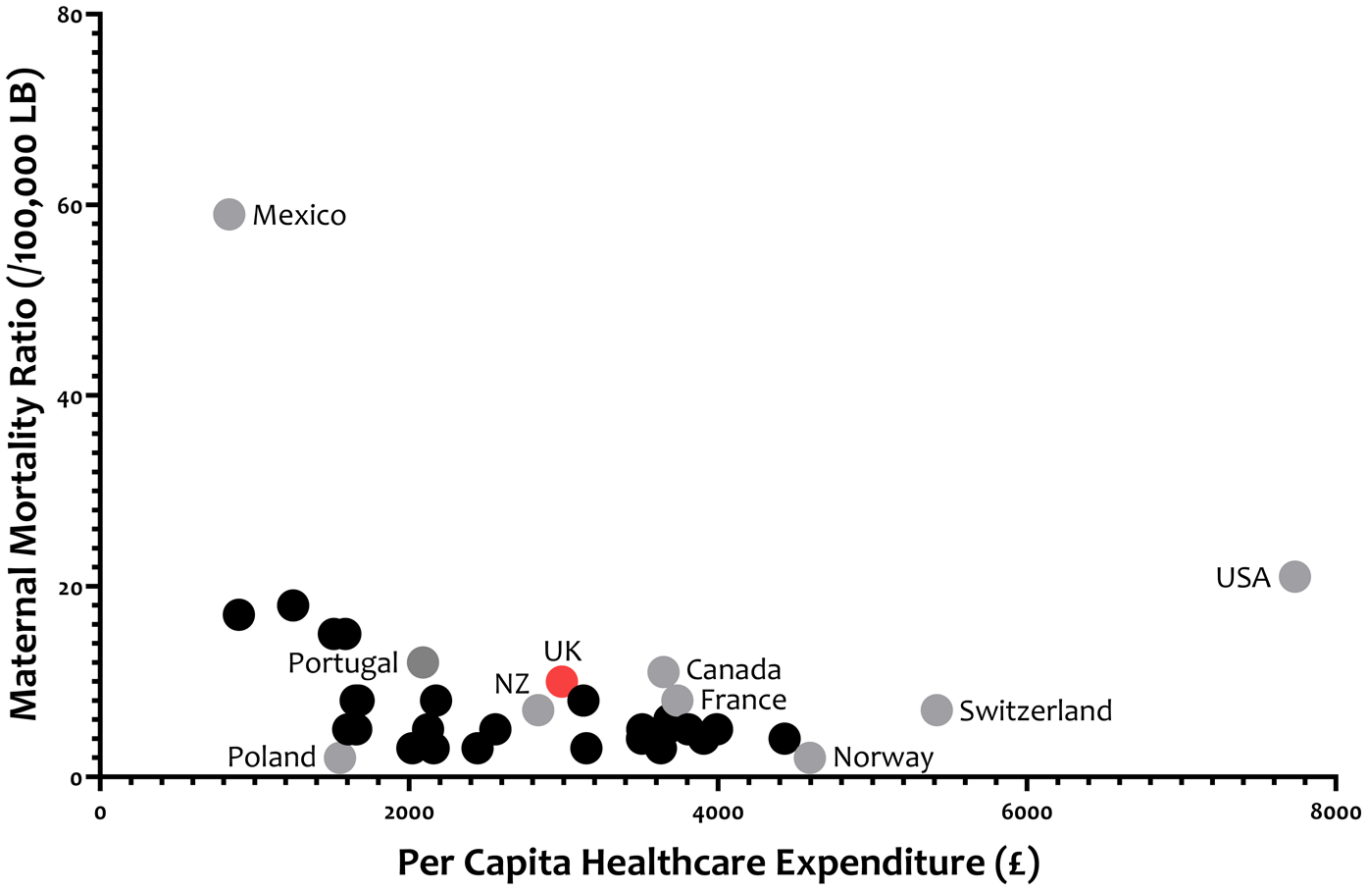
Per capita healthcare expenditure and MMR (The Global Health Observatory. Maternal mortality ratio (per 100 000 live births). https://wwwwhoint/data/gho/indicator‐metadata‐registry/imr‐details/3140 [accessed 11 Nov 2024]). The dots represent individual countries. Those in grey are labelled to provide further context. MMR, maternal mortality ratio; NZ, New Zealand; UK, United Kingdom; USA, United States of America.

The strategy outlined requires de‐implementation of the current, complex, antenatal *and* postnatal scoring systems, and implementation of a simplified and evidence‐based approach. Such a revised strategy would recommend less LMWH use, and be a source of anxiety for many clinicians and women. Many will call for more (not less) LMWH use, citing the key contribution of VTE‐related maternal mortality to overall rates in the UK [1]. This may reflect inadequate performance of currently‐used RAMS which guide thromboprophylaxis recommendations, or secular trends in VTE risk factors (e.g., more advanced maternal age, medical co‐morbidities, and elevated body weight complicating pregnancy) which have resulted in population characteristics that differ from those in which a RAM was developed [6]. Alternatively, rising VTE‐related maternal mortality may reflect the current challenges faced by NHS maternity services, and those in comparable health systems, such as Canada and New Zealand (Figure 2). An isolated focus on VTE runs the risk of being among ‘simplistic responses to deep problems’ [14].

It will be important to explore barriers to de‐implementation of the current VTE risk assessment algorithms and implementation of a revised strategy, which will be complex and require education and monitoring of process and clinical outcomes. Education for care‐providers must acknowledge decision‐related anxiety and safety, whilst using evidence to build a compelling case for change. Education for care‐users should empower them to recognise potential VTE risk factors alongside those that reduce risk, as well as symptoms and signs of potential VTE (prompting diagnostic testing). Planned change must occur in manageable steps, associated with reassurance and support, whilst priming clinicians for such a change in practice and addressing clinician concern. Champions for change will be critical. Tracking progress and providing constructive feedback must build trust and foster an environment ready to adapt to new evidence and future, ongoing change. Monitoring is essential to track that: a RAM can be applied consistently and accurately; VTE and VTE‐related maternal mortality do not increase; clinicians' and women's experiences are monitored and fed back; radiological investigations for suspected VTE do not increase; and costs are assessed.

There are busy times ahead.

## Author Contributions

All authors participated and played a role in the conception and planning of this work. L.A.M. wrote the first draft. All authors contributed to the review and revision.

## Ethics Statement

No ethics approval was required for the stakeholder consultation that informed this commentary.

## Conflicts of Interest

The authors declare no conflicts of interest.

## Data Availability

Data sharing not applicable to this article as no datasets were generated or analysed during the current study.
